# RBPTD: a database of cancer-related RNA-binding proteins in humans

**DOI:** 10.1093/database/baz156

**Published:** 2020-02-11

**Authors:** Kun Li, Zhi-Wei Guo, Xiang-Ming Zhai, Xue-Xi Yang, Ying-Song Wu, Tian-Cai Liu

**Affiliations:** Institute of Antibody Engineering, School of Laboratory Medicine and Biotechnology, Southern Medical University, 1838 N. Guangzhou Ave, Guangzhou, 510515, China

**Keywords:** cancer, CNV, differential expression, RNA binding protein, survival analysis

## Abstract

RNA-binding proteins (RBPs) play important roles in regulating the expression of genes involved in human physiological and pathological processes, especially in cancers. Many RBPs have been found to be dysregulated in cancers; however, there was no tool to incorporate high-throughput data from different dimensions to systematically identify cancer-related RBPs and to explore their causes of abnormality and their potential functions. Therefore, we developed a database named RBPTD to identify cancer-related RBPs in humans and systematically explore their functions and abnormalities by integrating different types of data, including gene expression profiles, prognosis data and DNA copy number variation (CNV), among 28 cancers. We found a total of 454 significantly differentially expressed RBPs, 1970 RBPs with significant prognostic value, and 53 dysregulated RBPs correlated with CNV abnormality. Functions of 26 cancer-related RBPs were explored by analysing high-throughput RNA sequencing data obtained by crosslinking immunoprecipitation, and the remaining RBP functions were predicted by calculating their correlation coefficient with other genes. Finally, we developed the RBPTD for users to explore functions and abnormalities of cancer-related RBPs to improve our understanding of their roles in tumorigenesis.

Database URL: http: //www.rbptd.com

## Introduction

RNA-binding proteins (RBPs) are proteins that combine with RNA to form protein complexes to regulate RNA expression and exert its functions in physiological and pathological processes (1, 2). RBPs can regulate gene expression by different ways. For instance, it can regulate gene expression at the post-transcriptional level by affecting RNA stability and transportation (3–8). In addition, it also regulates gene expression by directing ribosomes to affect the rate of protein synthesis at the translational level (9). Therefore, the normal RBPs play important roles in human cellular processes.

RBPs are closely associated with a variety of human diseases, especially cancers (5, 6, 10–14). Substantial studies have revealed that dysregulated RBPs have been proven to play essential roles in tumorigenesis (15, 16). For example, RBP U2AF1 affects pre-mRNA splicing of a large number of known oncogenic drivers to promote tumorigenesis (17). Therefore, it is essential to integrate the expression profiles of RBPs to systematically investigate their functions in cancers.

**Figure 1:**
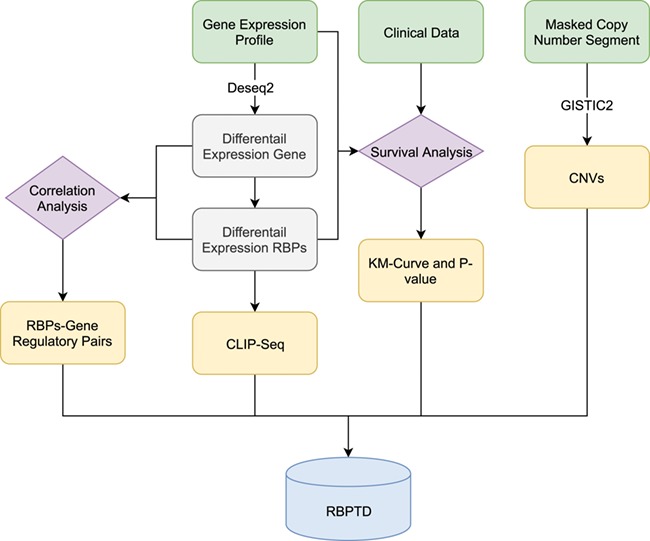
Workflow of RBPTD development. Expression profiles, prognostic information and copy number variation (CNV) data were downloaded from The Cancer Genome Atlas (TCGA). To find the cancer-related RBPs, we used the DESeq2 package in the R software environment to analyse differentially expressed genes (DEGs), and the survival package of R software was used for survival analysis. To reveal the causes of abnormality of dysregulated RBPs, the genomic identification of significant targets in cancer (GISTIC) was used for CNV analysis. We also predicted the function of RBPs by CLIP-Seq experimental data and co-expression analysis. Finally, we then developed a database using the obtained RNA binding protein (RBP) results.

**Figure 2:**
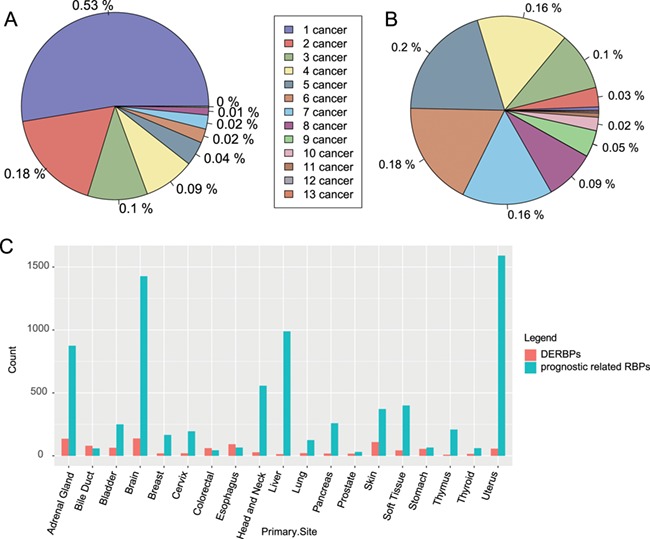
Distribution of RBPs among cancers. (**A**) Distribution of significantly differentially expressed RBPs among cancers. (**B**) Distribution of RBPs with significant prognostic value among caners. (**C**) Count of DERBPs and RBPs with prognostic values in cancers. The number ahead of cancer means the number of cancers with the same RBPs. DERBPs = differentially expressed RBPs.

Previous studies have revealed that DNA copy number variation (CNV) has a significant impact on gene expression regulation and is involved in tumorigenesis (18, 19). Therefore, the identification of CNV-related dysregulation of RBPs may reveal the causes of abnormality of some dysregulated RBPs. Since RBPs mainly exert their functions by combining with transcripts, high-throughput experimental methods, such crosslinking immunoprecipitation sequencing (CLIP-Seq), can be used to identify their target sites in gene transcripts. Thus, it is important to integrate multiple types of data to investigate their potential functions and the causes of abnormality.

To systematically investigate the functions of RBPs in cancers, we first identified cancer-related RBPs, including significantly differentially expressed RBPs and those with significant prognostic value, by analysing their gene expression profiles and prognostic information. We then explored their potential functions by analysing CLIP-Seq data for 26 RBPs and calculating the correlation of expression among the remaining RBPs with other genes. Finally, we investigated CNV-related dysregulated RBPs and developed a database to manage and present the results for reference.

## Methods

The analytical workflow of this study consists of four sections: cancer-related RBPs identification, CNV-analysis, function prediction and database construction (Figure 1).

### Identification of cancer-related RBPs

RBPs were identified by experimental methods including CLIP-Seq and computational prediction methods (20) following previously established methods (6, 21, 22). Gene expression profiles of 28 types of cancers were downloaded from The Cancer Genome Atlas (TCGA). Significantly differentially expressed RBPs were identified by comparing their expression levels in cancer tissues with those in adjacent normal tissues using the DESeq2 (ver. 3.7) with the default settings (23, 24). The raw *P* values were then adjusted to the false discovery rate (FDR) using the Benjamini–Hochberg procedure. Significant differential expression was assessed at a level of FDR ≤ 0.05. To identify RBPs with significant prognostic value, patients of each cancer type were divided into two groups according to mean RBP expression levels. The Kaplan–Meier method was then applied to determine differences in survival curves using the survival (25) package (ver. 2.38) in the R software environment. Significance was determined at a level of *P* value ≤0.05.

**Table 1:** Statics of cancer-related RBPs

Primary site	SUR	DE	CNV	DE & SUR	DE & CNV	DE & CNV & SUR
Adrenal gland	886	38	33	96	6	3
Bile duct	98	36	5	4	0	0
Bladder	300	25	87	18	2	2
Blood	843	0	0	0	0	0
Bone marrow	286	0	22	0	0	0
Brain	1455	84	90	128	5	4
Breast	206	14	173	7	0	0
Cervix	243	0	177	1	1	0
Colorectal	97	59	84	17	3	0
Eye	314	0	223	0	0	0
Head and neck	604	24	101	8	1	0
Kidney	697	0	124	0	0	0
Liver	1043	9	71	7	1	0
Lung	161	20	114	7	2	1
Lymph nodes	69	0	24	0	0	0
Nervous system	418	0	0	0	0	0
Oesophagus	90	55	50	0	2	0
Ovary	79	0	98	0	0	0
Pancreas	297	0	85	6	2	0
Pleura	353	0	68	0	0	0
Prostate	69	16	86	4	1	0
Skin	420	11	185	34	15	4
Soft tissue	445	0	117	6	2	0
Stomach	96	50	66	10	2	1
Testis	25	0	39	0	0	0
Thymus	240	1	13	3	1	0
Thyroid	113	13	0	0	0	0
Uterus	1635	47	172	46	7	6
**Total**	**1970**	**454**	**975**	**272**	**45**	**21**

Numbers of significant RNA binding proteins (RBPs), categorised by survival (SUR), differential expression (DE) and copy number variants (CNV). Boolean operator (&) indicates RBPs common among multiple categories.

### CNV analysis

To identify CNV-related dysregulated RBPs among 28 types of cancer, the copy number segment of each cancer patient was downloaded from TCGA. These data were then analysed using the genomic identification of significant targets in cancer (GISTIC) (ver. 2.0) (26) with the human hg38 reference genome. According to the G-score calculated by GISTIC, significantly amplified or deleted regions were identified for each patient. The false discovery rate (FDR) was then calculated for aberrant regions, and regions with FDR ≤ 0.05 were identified (26–28).

### Function prediction

RBPs predominantly exert their functions by combining with RNA to affect their stability or functions. The CLIP-Seq data of RBPs were applied to identify RBP binding sites in gene transcripts obtained from the StarBase (ver. 3.0) (29, 30) and the CLIP-Seq of 26 cancer-related RBPs were found. In addition, Pearson correlation coefficients between RBPs and other genes were calculated using the Hmisc package in R to predict their potential targets. Putative regulatory pairs with coefficients ≥0.4 and *P* ≤ 0.05 were retained.

### Database construction

The RBPTD database was constructed based on the Apache (ver. 2.0), PHP (ver. 7) and MySQL (ver. 5.3) software packages. We used the Vue Cli and BootstrapVue tools as a framework for building user interfaces. The gene expression boxplot and Kaplan–Meier survival curves were provided by the ECharts charting library.

## Results

### Identification of cancer-related RBPs

Our comparison of gene expression profiles between cancer tissues and adjacent normal tissues across 28 cancer types identified 454 significantly differentially expressed RBPs (|log_2_ fold change| ≥1 and FDR ≤ 0.05). Among these, 236 RBPs were cancer type-specific, and 218 were differentially expressed in more than one cancer type (Figure 2A). Among the significantly differentially expressed RBPs found in multiple cancer types, CPEB1 was differentially expressed in nine types of cancer; previous studies have reported its ability of promoting tumour migration in breast (31), liver (32) and endometrial cancers (33).

Our analysis of the prognosis of RBPs in 26 types of cancer identified 1970 RBPs with significant prognostic value; of these, 218 (approximately 11%) were found in more than one type of cancer (Figure 2B).

### Causes and functions of dysregulated RBPs

To explore the potential functions of RBPs, we predicted gene transcript target sites using CLIP-Seq. We found a total of 97 105 binding sites among 26 RBPs in the experimental data. Because the amount of available CLIP-Seq data was limited, we predicted the regulated genes of remaining RBPs by calculating their correlation coefficient with other genes. In total, we found 26 030 969 regulated RBP–gene pairs, involving 1924 RBPs and 19 564 putative target genes (Table 1).

**Figure 3:**
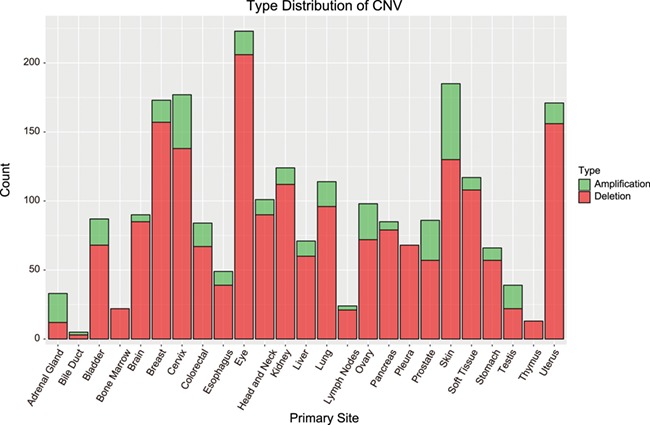
Distribution of CNVs among cancer types. Bar height indicates the number of genes with CNV in the primary site. Amplification = amplified regions. Deletion = deleted regions.

Apart from exploring RBP functions, we also identified dysregulated RBPs caused by DNA amplification and deletion. By analysis of CNV-related dysregulated RBPs using GISTIC, we found that 2307 CNVs in 975 RBPs (*P* value ≤0.05 and FDR≤0.05; Table 1). These CNVs included 367 amplifications in 243 genes and 1938 deletions in 828 cancer-related RBPs (Figure 3).

### Database interface

**Figure 4:**
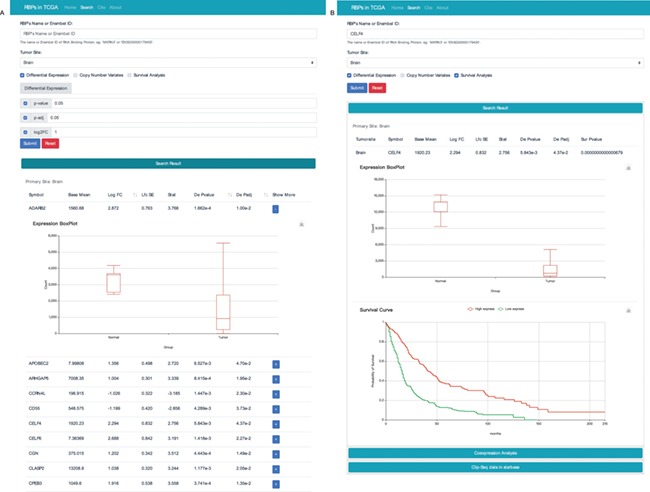
RBP result interface with two search modes. (**A**) Search the database according to tumour site, *P* value, p-adj and/or log_2_FC. (**B**) Search the database according to the name of RBP and cancer type. To get more details, users can click the “+” button.

We constructed a website to search and display our integrated data. The website mainly consists of four sections. The search interface can be used to search for RBPs in one or more cancer types with various filter options, such as gene expression fold change, prognostic value and change in CNV (Figure 4A). The web interface also allows users to search the database by RBP name or ensemble identifier (Figure 4B).

## Discussion

RBPTD is a database for the systematic investigation of RBP functions and abnormal causes of cancer-related RBPs. The database recognises 1991 cancer-related RBPs including 454 significantly differentially expressed RBPs and 1970 RBPs with significant prognostic value among 28 types of cancer. To explore their functions, target sites of 26 RBPs were identified in this study using CLIP-Seq data. Besides, we found 26 039 691 significantly related RBP-gene regulatory pairs for the remaining RBPs by calculating their Pearson correlation coefficients. Finally, we found 45 CNV-related dysregulated RBPs.

This study is the first study to incorporate multiple high-throughput data from different dimensions to systematically investigate cancer-related RBPs. Previous studies mainly focused on specific aspect of RBPs, such as dysregulated RBPs identification and SNP-related RBPs (15, 16, 34). In our study, we predicted cancer-related RBPs by the analysis of differential gene expression, prognosis and CNV. In addition, we not only explored the functions of RBPs by analysing CLIP-Seq data and calculating their correlation with other genes, but also investigated the causes of abnormality for the dysregulated RBPs.

There were several limitations to the current study. Some types of cancer have been reported without control datasets; therefore, significantly differentially expressed genes could not be identified for these types of cancer. In addition, the limited number of CLIP-Seq data limited the function prediction of RBPs. The function prediction based on Pearson correlation may be false positive, and have no experimental data for support.

In this study, we developed RBPTD to systematically identify cancer-related RBPs, predict their potential functions in cancers and explore causes of abnormalities among significantly differentially expressed RBPs. We expect that our database will promote further understanding of the roles of RBPs in tumorigenesis.

## References

[1] Almal S.H. and Padh H. (2012) Implications of gene copy-number variation in health and diseases. J. Hum. Genet., 57, 6–13. 21956041 10.1038/jhg.2011.108

[2] Feuk L., Carson A.R. and Scherer S.W. (2006) Structural variation in the human genome. Nat. Rev. Genet., 7, 85–97. 16418744 10.1038/nrg1767

[3] Pereira B., Billaud M. and Almeida R. (2017) RNA-binding proteins in cancer: old players and new actors. Trends Cancer, 3, 506–528. 28718405 10.1016/j.trecan.2017.05.003

[4] Zhou B. and Guo R. (2018) Integrative analysis of significant RNA-binding proteins in colorectal cancer metastasis. J. Cell. Biochem., 119, 9730–9741. 30132996 10.1002/jcb.27290

[5] Lukong K.E., Chang K., Khandjian E.W. et al. (2008) RNA-binding proteins in human genetic disease. Trends Genet., 24, 416–425. 18597886 10.1016/j.tig.2008.05.004

[6] Castello A., Fischer B., Hentze M.W. et al. (2013) RNA-binding proteins in Mendelian disease. Trends Genet., 29, 318–327. 23415593 10.1016/j.tig.2013.01.004

[7] Gerstberger S., Hafner M. and Tuschl T. (2014) A census of human RNA-binding proteins. Nat. Rev. Genet., 15, 829–845. 25365966 10.1038/nrg3813 PMC11148870

[8] Oliveira C., Faoro H., Alves L.R. et al. (2017) RNA-binding proteins and their role in the regulation of gene expression in Trypanosoma cruzi and Saccharomyces cerevisiae. Genet Mol. Biol., 40, 22–30. 28463381 10.1590/1678-4685-GMB-2016-0258 PMC5409782

[9] Nishida K., Kuwano Y., Nishikawa T. et al. (2017) RNA binding proteins and genome integrity. Int. J. Mol. Sci., 18, E1341. 28644387 10.3390/ijms18071341 PMC5535834

[10] Belanger K., Nutter C.A., Li J. et al. (2018) CELF1 contributes to aberrant alternative splicing patterns in the type 1 diabetic heart. Biochem. Biophys. Res. Commun., 503, 3205–3211. 30158053 10.1016/j.bbrc.2018.08.126 PMC6142808

[11] Avdulov S., Li S., Michalek V. et al. (2004) Activation of translation complex eIF4F is essential for the genesis and maintenance of the malignant phenotype in human mammary epithelial cells. Cancer Cell, 5, 553–563. 15193258 10.1016/j.ccr.2004.05.024

[12] Correa A.G.D., Lemos B.H.V., Nascimento M. et al. (2016) AR musical app for children’s musical education. 2016 IEEE International Symposium on Consumer Electronics (ISCE), 125–126.

[13] Musunuru K. (2003) Cell-specific RNA-binding proteins in human disease. Trends Cardiovas. Med., 13, 188–195. 10.1016/s1050-1738(03)00075-6 12837581

[14] Sebestyén E., Singh B., Miñana B. et al. (2016) Large-scale analysis of genome and transcriptome alterations in multiple tumors unveils novel cancer-relevant splicing networks. Genome Res., 26, 732–744. 27197215 10.1101/gr.199935.115 PMC4889968

[15] Wang J., Liu Q. and Shyr Y. (2015) Dysregulated transcription across diverse cancer types reveals the importance of RNA-binding protein in carcinogenesis. BMC Genomics, 16, S5. 10.1186/1471-2164-16-S7-S5 PMC4474540 26100984

[16] Kechavarzi B. and Janga S.C. (2014) Dissecting the expression landscape of RNA-binding proteins in human cancers. Genome Biol., 15, R14. 24410894 10.1186/gb-2014-15-1-r14 PMC4053825

[17] Okeyo-Owuor T., White B.S., Chatrikhi R. et al. (2015) *U2AF1* mutations alter sequence specificity of pre-mRNA binding and splicing. Leukemia, 29, 909–917. 25311244 10.1038/leu.2014.303 PMC4391984

[18] Dweep H., Kubikova N., Gretz N. et al. (2015) Homo sapiens exhibit a distinct pattern of CNV genes regulation: an important role of miRNAs and SNPs in expression plasticity. Sci. Rep., 5, 12163. 26178010 10.1038/srep12163 PMC4503977

[19] Mehta D., Iwamoto K., Ueda J. et al. (2014) Comprehensive survey of CNVs influencing gene expression in the human brain and its implications for pathophysiology. Neurosci. Res., 79, 22–33. 24211644 10.1016/j.neures.2013.10.009

[20] Si J., Cui J., Cheng J. et al. (2015) Computational prediction of RNA-binding proteins and binding sites. Int. J. Mol. Sci., 16, 26303–26317. 26540053 10.3390/ijms161125952 PMC4661811

[21] Ray D., Kazan H. and Cook K.B. (2013) A compendium of RNA-binding motifs for decoding gene regulation. Nature, 499,172–177. 23846655 10.1038/nature12311 PMC3929597

[22] Castello A., Fischer B., Frese C.K. et al. (2016) Comprehensive identification of RNA-binding domains in human cells. Mol. Cell, 63, 696–710. 27453046 10.1016/j.molcel.2016.06.029 PMC5003815

[23] Love M.I., Huber W. and Anders S. (2014) Moderated estimation of fold change and dispersion for RNA-seq data with DESeq2. Genome Biol., 15, 550. 25516281 10.1186/s13059-014-0550-8 PMC4302049

[24] Fu J, Frazee AC, Collado-Torres L, et al Ballgown: flexible, isoform-level differential expression analysis. R package version 2 12 0.

[25] Gray R.J. (2002) Modeling survival data: extending the Cox model. J. Am. Stat. Assoc., 97, 353–354.

[26] Mermel C.H., Schumacher S.E., Hill B. et al. (2011) GISTIC2.0 facilitates sensitive and confident localization of the targets of focal somatic copy-number alteration in human cancers. Genome Biol., 12, R41. 21527027 10.1186/gb-2011-12-4-r41 PMC3218867

[27] Beroukhim R., Mermel C.H., Porter D. et al. (2010) The landscape of somatic copy-number alteration across human cancers. Nature, 463, 899–905. 20164920 10.1038/nature08822 PMC2826709

[28] Beroukhim R., Getz G., Nghiemphu L. et al. (2007) Assessing the significance of chromosomal aberrations in cancer: methodology and application to glioma. Proc. Natl. Acad. Sci. U.S.A., 104, 20007–20012. 18077431 10.1073/pnas.0710052104 PMC2148413

[29] Li J.-H., Liu S., Zhou H. et al. (2014) starBase v2.0: decoding miRNA-ceRNA, miRNA-ncRNA and protein–RNA interaction networks from large-scale CLIP-Seq data. Nucleic Acids Res., 42, D92–D97. 24297251 10.1093/nar/gkt1248 PMC3964941

[30] Yang J.-H., Li J.-H., Shao P. et al. (2011) starBase: a database for exploring microRNA–mRNA interaction maps from Argonaute CLIP-Seq and Degradome-Seq data. Nucleic Acids Res., 39, D202–D209. 21037263 10.1093/nar/gkq1056 PMC3013664

[31] Nagaoka K., Fujii K., Zhang H. et al. (2016) CPEB1 mediates epithelial-to-mesenchyme transition and breast cancer metastasis. Oncogene, 35, 2893–2901. 26411364 10.1038/onc.2015.350 PMC4809797

[32] Xu M., Fang S., Song J. et al. (2018) CPEB1 mediates hepatocellular carcinoma cancer stemness and chemoresistance. Cell Death Dis., 9, 957. 30237545 10.1038/s41419-018-0974-2 PMC6148052

[33] Xiong H., Chen R., Liu S. et al. (2018) MicroRNA-183 induces epithelial–mesenchymal transition and promotes endometrial cancer cell migration and invasion in by targeting CPEB1. J. Cell. Biochem., 119, 8123–8137. 29923214 10.1002/jcb.26763

[34] Neelamraju Y., Gonzalez-Perez A., Bhat-Nakshatri P. et al. (2017) Mutational landscape of RNA-binding proteins in human cancers. RNA Biol., 15, 115–129. 29023197 10.1080/15476286.2017.1391436 PMC5786023

